# Nurse‐Led Screening‐Triggered Early Specialized Palliative Care Program for Patients With Advanced Lung Cancer: A Multicenter Randomized Controlled Trial

**DOI:** 10.1002/cam4.70325

**Published:** 2024-11-18

**Authors:** Yoshihisa Matsumoto, Shigeki Umemura, Ayumi Okizaki, Daisuke Fujisawa, Takuhiro Yamaguchi, Shunsuke Oyamada, Tempei Miyaji, Tomoe Mashiko, Naoko Kobayashi, Eriko Satomi, Daisuke Kiuchi, Tatsuya Morita, Yosuke Uchitomi, Koichi Goto, Yuichiro Ohe

**Affiliations:** ^1^ Department of Palliative Medicine National Cancer Center Hospital East Kashiwa Japan; ^2^ Department of Palliative Therapy Cancer Institute Hospital of Japanese Foundation for Cancer Research Tokyo Japan; ^3^ Department of Thoracic Oncology National Cancer Center Hospital East Kashiwa Japan; ^4^ Center for Public Health Sciences National Cancer Center Tokyo Japan; ^5^ Innovation Center for Supportive Palliative and Psychosocial Care, National Cancer Center Hospital Tokyo Japan; ^6^ Department of Neuropsychiatry Keio University School of Medicine Tokyo Japan; ^7^ Division of Biostatistics Tohoku University School of Medicine Sendai Japan; ^8^ Department of Biostatistics JORTC Data Center Tokyo Japan; ^9^ Department of Clinical Trial Data Management Graduate School of Medicine, the University of Tokyo Tokyo Japan; ^10^ Department of Nursing National Cancer Center Hospital East Kashiwa Japan; ^11^ Department of Palliative Medicine National Cancer Center Hospital Tokyo Japan; ^12^ Department of Palliative Care Center Hospital of the National Center for Global Health and Medicine Tokyo Japan; ^13^ Palliative and Supportive Care Seirei Mikatahara Hospital Hamamatsu Japan; ^14^ Department of Thoracic Oncology National Cancer Center Hospital Tokyo Japan

**Keywords:** advanced lung cancer, nurse‐led program, palliative care, quality of life, randomized controlled trial, screening

## Abstract

**Background:**

We aimed to examine the effectiveness of a nurse‐led, screening‐triggered early specialized palliative care intervention program for patients with advanced lung cancer.

**Methods:**

Patients with advanced lung cancer who underwent initial chemotherapy were randomized to intervention and usual care groups between January 2017 and September 2019. The intervention comprised comprehensive needs assessments, counseling, and service coordination by advanced‐level nurses. Patients in the usual care group received the usual oncological care. The primary end point was a change in the trial outcome index (TOI) scores from baseline to 12 weeks. The secondary end‐points were TOI scores at week 20, depression, anxiety, and survival.

**Results:**

In total, 102 patients were assigned to each group. Compared with the usual care group, no significant improvement in TOI scores was observed at 12 weeks in the intervention group (mean group difference: 2.13; 90% confidence interval: −0.70, 4.95; *p* = 0.107, one‐sided), whereas significant improvement was observed at 20 weeks (3.58; 90% confidence interval: 0.15, 7.00; *p* = 0.043). There were no significant differences in the change from baseline depression and anxiety between the groups from baseline at week 12 and 20 weeks (depression: *p* = 0.60 and 0.10, anxiety: *p* = 0.78 and 0.067). Survival did not significantly differ between the groups (median survival time: 12.1 vs. 11.1 months; *p* = 0.302).

**Conclusions:**

Nurse‐led, screening‐triggered, early specialized palliative care did not show significant superiority over usual care during the 12‐week study period. However, it may have yielded delayed clinical benefits, such as improved quality of life and this feasible model can be acceptable in clinical practice.

**Trial Registration:** The University Hospital Medical Information Network Clinical Trials Registry: UMIN000025491

## 1 Introduction

Early palliative care (EPC) integrated with standard oncological care helps relieve patients' symptoms, thereby improving their quality of life (QOL) [1]. Studies have shown the positive effects of EPC on QOL and other outcomes in patients with advanced cancer [2, 3, 4, 5, 6]. However, findings have been inconsistent due to different models of EPC being used [7, 8, 9]. EPC was efficacious when delivered by palliative care specialists to all patients from the first contact but did not demonstrate significant effects when administered by advanced‐level nurses as primary palliative care [7]. Given that the former approach is costly and only feasible in facilities with abundant medical resources, establishing a more practical and cost‐effective EPC model is necessary [10]. Theoretically, distress screening is a potential solution for programs with limited human resources; however, its effectiveness has not been confirmed [11, 12, 13]. Moreover, factors accounting for the beneficial effects of EPCs remain unknown. Improvement in patient perception of illness, discussions between clinicians and patients regarding coping strategies, and clinician support for patients' decision‐making are presumed to contribute to the effectiveness of EPC, although this has not been proven yet [14, 15]. Studies are warranted to identify the core components of EPC interventions [16]. Additionally, since the effectiveness of palliative care services may be influenced by sociocultural factors and the healthcare system where they are provided, it is important to develop a practical conceptual model tailored to the current conditions.

We devised a novel nurse‐led, screening‐triggered, early specialized palliative care intervention program for delivering specialized palliative care by combining screening with a stepped‐care approach. The novelty of this strategy lies in promoting specialized palliative care intervention for patients with potential needs as a priority through screening and employing stepped intervention, which transitions from advanced‐level nurses to an interdisciplinary team when needed. We previously examined the feasibility of this intervention in 50 patients with advanced lung cancer using a single‐arm, pre–post‐design study, and we observed improved QOL and psychological status among the participants [17]. This study aimed to examine the effectiveness of our novel palliative care intervention program in patients with advanced lung cancer.

## 2 Methods

### 2.1 Study Design

This study was a multicenter, parallel‐group, randomized controlled trial conducted at two comprehensive cancer centers in Japan: the National Cancer Center East in Kashiwa and the National Cancer Center Hospital in Tokyo. We recruited patients with newly diagnosed advanced lung cancer. Following the UK Medical Research Council's recommendation, we employed a mixed‐method approach for analysis, setting multiple secondary end‐points and conducting a qualitative analysis [18]. This study was approved by the Institutional Review Board of the National Cancer Center, Japan (approval number: 2016‐235). The study protocol details have been described previously [19].

### 2.2 Patients

The eligibility criteria for study participation included the following: (1) pathologically or cytologically confirmed diagnosis of lung cancer; (2) diagnosis of stage IV non‐small cell lung cancer (NSCLC) or extensive disease small cell lung cancer (SCLC); (3) negative or unknown status of gene mutations, such as EGFR, ALK, ROS1, or BRAF, for which molecular targeted therapy was applicable; (4) scheduled for first‐line chemotherapy (excluding immunotherapy); (5) no previous treatment for lung cancer, including chemotherapy, surgery, radiation therapy with curative intent, and/or immunotherapy; (6) initial administration of first‐line chemotherapy in an inpatient setting (second and subsequent administration could be given either in an inpatient or an outpatient setting); (7) age ≥ 20 years; and (8) willingness to provide written informed consent. Participants were excluded if they (1) had received specialized palliative care interventions (including psycho‐oncology care), (2) had severe cognitive impairment, (3) were unable to comprehend Japanese, (4) were already participating in other interventional studies that prohibited participation in the current research, or (5) were considered ineligible for this study by their attending physician.

### 2.3 Random Assignment

The research staff approached eligible patients and obtained written informed consent. Next, they were randomly assigned to either the intervention group (nurse‐led, screening‐triggered palliative care intervention) or the control group (usual oncological care) at a 1:1 ratio. Randomization was performed using a stratified permuted block method with a computer random number generator. The allocation was stratified by (1) histological type of cancer (NSCLC or SCLC), (2) study site, and (3) patient age (< 75 or ≥ 75 years). Blinding was not possible owing to the nature of the intervention.

### 2.4 Intervention Group

Patients in the intervention group received nurse‐led, screening‐triggered, specialized palliative care, which included the following components:

#### 2.4.1 Screening

The intervention started with a brief screening questionnaire, which is completed after study enrollment and before initial administration of first‐line chemotherapy. This self‐administered questionnaire covered four domains (physical, psychological, social, and medical/information needs). Physical distress was assessed using a single question adapted from the physical domain of the Support Team Assessment Schedule [20], and graded using a 5‐point Likert scale (ranging from 0 = no physical distress to 4 = persistent unendurable physical distress). A score of ≥ 2 indicated physical distress. The psychological domain corresponded to the distress and interference thermometer, a widely used screening tool in the Japanese cancer population [21, 22]. This scale comprises an item that assesses the level of psychological distress on a thermometer‐shaped numeric scale (ranging from 0 = no distress to 10 = extreme distress) and another item assessing the level of interference with daily life activities resulting from distress (ranging from 0: no interference to 10: extreme interference). A distress score of ≥ 4 and an interference score of ≥ 3 indicated psychological distress [21]. The presence of social distress was evaluated by a single question: “Do you currently have any concern about financial issues, employment issues, or any other issues in daily living?” The options included “Yes,” “No current concern but want to talk with someone on these issues,” or “No concern at all.” The first two responses indicated social distress. The fourth domain of the questionnaire was designed to assess participants' need for additional information on their illness and/or treatment using the following question: “Do you currently have any concern, or do you have anything you want to know further about your illness and/or treatment?” The patients' needs related to their illnesses and/or treatments were communicated to the physician/nurse in charge through medical records.

#### 2.4.2 Advanced‐Level Nurse

The nurses involved in this intervention were required to (1) hold advanced‐level certification (certified nurse or certified nurse specialist) in the relevant fields and (2) have completed at least 10 h of training based on the intervention manual (available on request addressed to the corresponding author). Certified nurses are qualified nurses who have at least 5 years of clinical experience and have received at least 6 months of advanced‐level training in one of 21 specialized areas. Certified nurse specialists are master‐level nurses who have at least 5 years of clinical experience and have received at least 2 years of advanced‐level training in one of 10 specialized areas. Both of the credentials are authenticated by the Japanese Nursing Association. In the current study, certified nurses in palliative care, certified nurses in cancer pain management nursing, certified nurse specialists in cancer nursing, and certified nurse specialists in psychiatric mental health nursing are eligible for participation.

#### 2.4.3 Counseling and Care Coordination by an Advanced‐Level Nurse

A positive screening result for any of the physical distress, psychological distress, or socioeconomic need subscales from the abovementioned questionnaire prompted intervention by a specialized palliative care team. Initially, an advanced‐level nurse contacted the patient and conducted a comprehensive physical, psychological, social, and medical/informative assessment. During this process, the nurse attempted to provide the following care based on the findings of a previous palliative care study [14]: (1) building rapport, (2) managing symptoms, (3) helping the patients cope with cancer diagnosis, (4) facilitating patients' understanding of their illness and treatment, (5) counseling on anticancer treatment and its adverse effects, (6) preparing for cancer progression and end‐of‐life, and (7) facilitating family involvement. The advanced‐level nurse either directly counseled the patients or referred them to other professionals, such as a medical social worker for financial problems or a palliative care physician for physical symptoms.

#### 2.4.4 Interdisciplinary Team Approach

The care plan was regularly reviewed by an interdisciplinary palliative care team. Hospitalized patients were reviewed weekly by a team of palliative care physicians, nurses, psychiatrists, psychologists, social workers, pharmacists, and nutritionists.

For ambulatory patients, the plans were reviewed biweekly by an advanced‐level nurse and a board‐certified palliative care physician. Based on the findings, other professionals provided additional specialized palliative care.

#### 2.4.5 Follow‐Up

Once the intervention by the specialized palliative care team began, it continued for 5 months until the end of the study period. An advanced‐level nurse met with ambulatory patients at least once a month and hospitalized patients at least once a week. Patients who initially tested negative in the screening underwent the same brief screening every month, with a 3‐week margin. The intervention by the advanced‐level nurses was initiated only when the screening results were positive. However, the specialized palliative care team provided services upon request by patients, their families, or medical professionals. The participants continued to receive their usual oncological care during the study period.

### 2.5 Usual Care Group

Patients assigned to the usual care group received the usual oncological care. They did not undergo a brief screening and were not scheduled to meet the palliative care service team unless requested by the patient, their family, or the treating oncologists.

### 2.6 Measurements

#### 2.6.1 Primary Outcome

The primary outcome was the change in trial outcome index (TOI) scores from baseline to 12 weeks. The TOI score (84‐point scale) represented the physical condition and QOL of patients with lung cancer [23]. It was the sum of the scores of the physical and functional well‐being scores and lung cancer subscales (LCS) of the Functional Assessment of Cancer Therapy‐Lung (FACT‐L) which is a widely used scale that has been validated for its reliability in indicating the QOL of patients with lung cancer [23]. A change of 5–6 points in the TOI score was considered clinically meaningful [24].

#### 2.6.2 Secondary Outcomes

##### 2.6.2.1 Disease‐Specific QOL

We used the FACT‐L, a combination of the Functional Assessment of Cancer Therapy‐General (FACT‐G) and LCS, to evaluate QOL. The FACT‐L was self‐administered by patients at baseline, 3 months post‐randomization, and 5 months post‐randomization.

##### 2.6.2.2 Depression and Anxiety

We used the Patient Health Questionnaire‐9 (PHQ‐9), a 9‐item valid self‐reported instrument, to assess depression in patients [25]. Higher scores indicated more severe depression, with a total score of 10 indicated clinically significant depression. Patients were diagnosed with major depressive syndrome if they had at least five of the nine depression symptoms on the scale, with either anhedonia or depressed mood as one of them. We used the Generalized Anxiety Disorder‐7 (GAD‐7), a 7‐item valid self‐reported instrument, to measure anxiety levels [26]. A score of ≥ 10 out of 21 indicated clinically significant anxiety. The American Society of Clinical Oncology recommends both PHQ‐9 and GAD‐7 as screening tools to detect psychological distress in patients with cancer [27].

##### 2.6.2.3 Other Clinical Outcomes

We collected data on patient survival (1‐year survival rate and overall survival period), medical service use, and circumstances of death. The participants were followed up for 2 years after study enrollment.

### 2.7 Data Collection and Monitoring

Investigators maintained individual records for each patient as source data, including a copy of the informed consent, medical records, laboratory data, and other records or notes, while maintaining confidentiality. Patient‐reported outcome measures were accessed by paper questionnaires. All data were collected by the Japan Supportive, Palliative, and Psychosocial Oncology Group (J‐SUPPORT) Data Center at the Center for Public Health Science, National Cancer Center, Japan. The data management center supervised the intra‐study data sharing process. The REDCap electronic data capture application (Vanderbilt University) was used for patient enrollment, randomization, data entry, data management, and central monitoring [28]. Central data monitoring reports were compiled twice a year by clinical data managers and submitted to the principal and site investigators. No auditing was scheduled for this study.

### 2.8 Sample Size

We scheduled 206 participants to measure the primary outcome, which was the change in the TOI scores from baseline to 12 weeks between the intervention and control groups. Considering an estimated standard deviation (SD) score of 14 and an intraclass correlation of 0.6, along with an 80% power to detect a significant difference at the 5% alpha level (one‐sided) and an estimated attrition rate of 36% by week 12, the required sample size was calculated as 103 participants in each group. This calculation was based on a 5‐point difference in mean TOI scores, which was considered a clinically meaningful change [24]. A one‐sided test was used because it was not important to determine whether the intervention was significantly inferior to usual care.

### 2.9 Statistical Analyses

All randomized participants were included in statistical analyses. All statistical analyses were performed according to the predetermined statistical analysis plan. A general linear model with an unstructured covariance structure, including group, time point, time‐by‐group interaction, baseline TOI scores, and allocation factors as explanatory variables, was used to summarize the longitudinal change in TOI scores. We used all observed TOI scores from the baseline for each time point. For the primary end‐point, point estimates, and confidence intervals (Cis) for the mean change in TOI scores from baseline to 12 weeks were calculated and compared for both groups. Statistical tests were conducted for time‐by‐group interaction effects or (for models with only main effects if they are not significant) for group main effects. FACT‐L, LCS, PHQ‐9, and GAD‐7 scores were also analyzed alongside TOI scores. All analyses were determined in the intention‐to‐treat population. Survival was estimated by the Kaplan–Meier method and compared between groups using the log‐rank test. For the primary endpoint analysis, intervention effects were estimated with 90% CIs. For other analyses, 95% CIs were used. *p*‐values were one‐sided for the primary efficacy analysis and two‐sided for other analyses. A two‐sided significance level of 5% was considered statistically significant except for the primary analysis. Statistical analyses were performed using SAS software, version 9.4 (SAS Institute).

### 2.10 Ethical Consideration

This study was conducted in accordance with the principles of the Declaration of Helsinki and the Ethics Guideline for Clinical Studies of 2014 published by the Japan Ministry of Health, Labor and Welfare. Before enrollment, all patients provided written informed consent for study participation. The study was registered in the University Hospital Medical Information Network Clinical Trials Registry (UMIN000025491).

## 3 Results

### 3.1 Baseline Characteristics

**FIGURE 1:**
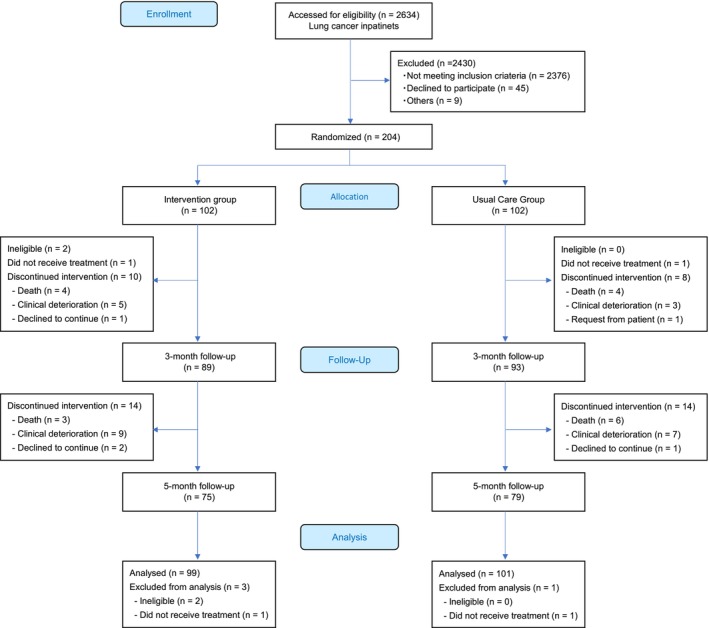
CONSORT flow diagram.

Figure 1 shows the CONSORT diagram, and Table 1 presents the baseline characteristics of the patients. From January 2017 to September 2019, we enrolled 204 patients (102 per group), with a mean age of 67.3 years (SD: 9.7 years), and 77.5% were men. While 72 patients had extensive disease SCLC, 132 had stage IV NSCLC. No other significant differences in baseline characteristics were observed between the two groups.

**TABLE 1:** Baseline characteristics of patients.

Characteristics	Intervention group (*n* = 102)		Usual care group (*n* = 102)	
	*n*	%	*n*	%
Mean age, years (SD)	67.1 (9.81)		67.5 (9.76)	
Male	79	77.5	79	77.5
Relationship status				
Married	71	69.6	80	78.4
Common‐law marriage	0	0.0	4	3.9
Unmarried	11	10.8	6	5.9
Divorced	9	8.8	5	4.9
Separation	0	0.0	1	1.0
Bereaved	11	10.8	6	5.9
Presence of coresidents				
Yes	85	83.3	90	88.2
Currently working				
Yes	50	49.0	44	43.1
Education				
High school or less	49	48.0	56	54.9
Vocational school or Junior college	14	13.7	16	15.7
4 or 6‐year university	31	30.4	26	25.5
Graduate school	0	0.0	2	2.0
Other	8	7.8	1	1.0
Smoking status				
Current smoker	3	2.9	5	4.9
Past smoker	91	89.2	89	87.3
Never smoker	8	7.8	8	7.8
Cancer type				
Non‐small cell lung cancer	66	64.7	66	64.7
Small cell lung cancer	36	35.3	36	35.3
ECOG PS				
0	45	44.1	37	36.3
1	45	44.1	56	54.9
2	10	9.8	6	5.9
3	2	2.0	3	2.9
Presence of history of malignancy				
Yes	10	9.8	16	15.7
No	92	90.2	86	84.3
Presence of history of mental illness				
Yes	4	3.9	1	1.0
No	98	96.1	101	99.0
Presence of comorbidities				
Yes	44	43.1	43	42.2
No	58	56.9	59	57.8
FACT‐L score, mean (SD)	86.1 (18.4)		90.2 (17.8)	
TOI score, mean (SD)	53.4 (14.2)		55.5 (14.1)	
LCS score, mean (SD)	17.0 (5.8)		17.5 (5.5)	
PHQ‐9 score, mean (SD)	6.3 (5.8)		6.1 (5.4)	
GAD‐7 score, mean (SD)	5.5 (5.4)		5.4 (5.1)	

Abbreviations: ECOG PS, Eastern Cooperative Oncology Group performance status; FACT‐L, functional assessment of cancer therapy‐lung; GAD‐7, generalized anxiety disorder‐7; PHQ‐9, patient health questionnaire‐9; SD, standard deviation; TOI, trial outcome index.

### 3.2 Intervention

Advanced‐level nurses initiated an intervention for 75 (73.5%), 9 (8.8%), 4 (3.9%), 3 (2.9%), and 1(1.0%) patients in the intervention group after completing the first, second, third, and fourth screening questionnaires, respectively. Two patients (2.0%) received an intervention without a positive screening result. Eight patients (7.8%) did not receive any intervention throughout the study. For the 94 patients in the intervention group, advanced‐level nurses conducted an average of 9.3 contacts (SD: 4.1 contacts) per patient, with an average contact time of 16.9 min (SD: 15.9 min) per contact. Conversely, in the usual care group, 43 patients (42.2%) had at least one encounter with a professional member of the palliative care team. The results of the brief questionnaire in the intervention group and the data on interventions by palliative care team members in the usual care group are provided in Tables S1 and S2.

### 3.3 QOL

**FIGURE 2:**
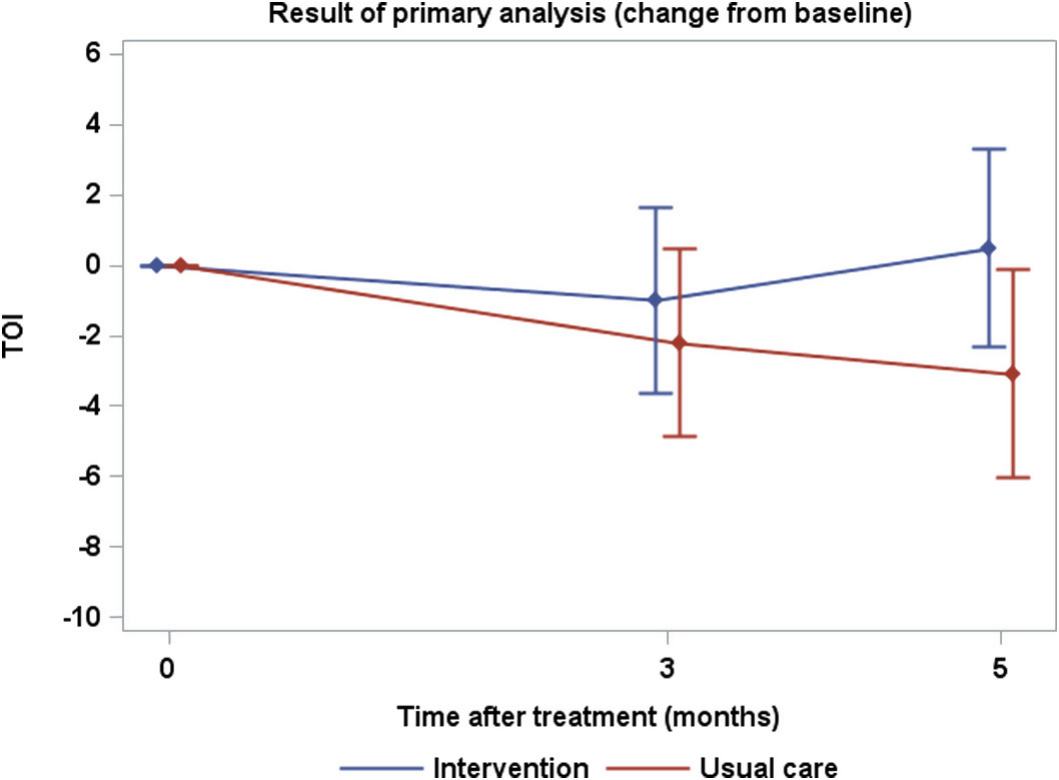
Result of primary analysis (Change from baseline). TOI, trial outcome index.

As there was no significant time‐by‐group interaction, we estimated the main effects. Compared with the usual care group, the intervention group showed no significant improvement in TOI scores from the baseline to week 12 (mean group difference [the same applies hereafter]: 2.13; 90% CI: −0.70, 4.95; *p* = 0.107, one‐sided) (Figure 2, Table 2). However, when considering time‐by‐group interaction effects, the intervention group showed a significant improvement in TOI scores from baseline to week 20 compared with the usual care group (3.58; 90% CI: 0.15, 7.00; *p* = 0.043). While the intervention group showed no significant improvement in FACT‐L scores at week 12, a significant improvement was observed at week 20 compared with the usual care group. However, the intervention group showed no significant improvement in LCS scores at weeks 12 and 20 compared with the usual care group.

**TABLE 2:** Scores and analysis of TOI, FACT‐L, LCS, PHQ‐9, and GAD‐7 for continuous data using general linear model (change from baseline).

			Intervention group				Usual care group				Group difference		*p*
			*n*	Mean (SD)	Estimates	CI^a^	*n*	Mean (SD)	Estimates	CI^a^	Estimates	CI^a^	
Primary													
TOI score													
Observed data	3 months		86	53.3 (15.5)			92	53.3 (15.4)					
	5 months		72	57.1 (14.8)			76	54.6 (16.2)					
Analysis for main effects					−0.41	−2.93 to 2.12			−2.53	−5.05 to −0.02	2.13	−0.70 to 4.95	0.107
Time‐by‐group interaction	Test for fixed‐effect												0.229
	3 months				−1.01	−3,65 to 1.62			−2.20	−4.86 to 0.45	1.19	−1.92 to 4.30	0.264
	5 months				0.50	−2.32 to 3.31			−3.08	−6.02 to −0.14	3.58	0.15 to 7.00	0.043
Secondary													
FACT‐L score													
Observed data	3 months		86	86.9 (20.5)			92	87.2 (19.8)					
	5 months		72	91.4 (19.6)			76	88.6 (21.3)					
Analysis for main effects					0.22	−2.96 to 3.40			−3.41	−6.43 to −0.39	3.62	−0.71 to 7.96	0.101
Time‐by‐group interaction	Test for fixed‐effect												0.767
	3 months				−0.72	−4.30 to 2.87			−2.87	−6.34 to 0.60	2.15	−2.81 to 7.12	0.394
	5 months				1.38	−2.35 to 5.11			−4.15	−7.94 to −0.37	5.53	0.27 to 10.80	0.040
LCS score													
Observed data	3 months		86	18.1 (5.8)			93	18.5 (5.5)					
	5 months		72	19.0 (4.8)			76	17.9 (5.8)					
Analysis for main effects					0.68	−0.27 to 1.62			0.18	−0.74 to 1.10	0.5	−0.82 to 1.82	0.458
Time‐by‐group interaction	Test for fixed‐effect												0.238
	3 months				0.60	−0.48 to 1.68			0.68	−0.32 to 1.67	−0.08	−1.55 to 1.39	0.916
	5 months				0.81	−0.16 to 1.79			−0.38	−1.54 to 0.78	1.19	−0.32 to 2.71	0.122
PHQ‐9 score													
Observed data	3 months		86	5.4 (5.9)			93	5.9 (5.8)					
	5 months		72	4.3 (5.1)			75	5.8 (5.7)					
Analysis for main effects					−0.67	−1.62 to 0.28			0.07	−0.82 to 0.97	−0.75	−2.06 to 0.56	0.262
Time‐by‐group interaction	Test for fixed‐effect												0.772
	3 months				−0.54	−1.60 to 0.52			−0.16	−1.10 to 0.78	−0.38	−1.81 to 1.05	0.602
	5 months				−0.89	−1.89 to 0.12			0.39	−0.75 to 1.53	−1.27	−2.79 to 0.25	0.101
GAD‐7 score													
Observed data	3 months		86	4.0 (5.1)			93	4.3 (5.4)					
	5 months		72	3.1 (4.6)			75	4.4 (4.7)					
Analysis for main effects					−1.39	−2.25 to −0.53			−0.75	−1.54 to 0.03	−0.644	−1.81 to 0.54	0.285
Time‐by‐group interaction	Test for fixed‐effect												0.534
	3 months				−1.25	−2.18 to −0.33			−1.07	−1.94 to −0.21	−0.18	−1.45 to 1.09	0.780
	5 months				−1.61	−2.60 to −0.62			−0.35	−1.26 to 0.57	−1.26	−2.61 to 0.09	0.067

Abbreviations: CI, confidence interval; standard deviation; FACT‐L, functional assessment of cancer therapy‐lung; GAD‐7, generalized anxiety disorder‐7; LCS, lung cancer subscale; PHQ‐9, patient health questionnaire‐9; TOI, trial outcome index.

^a^ The primary end point analysis shows 90% confidence intervals and the secondary end point analyses show 95% confidence intervals.

### 3.4 Depression and Anxiety

There was no significant difference in the change from baseline depression and anxiety between the two groups at week 12 (depression: −0.38; 95% CI: −1.81, 1.05; *p* = 0.602, anxiety: −0.18; 95% CI: −1.45, 1.09; *p* = 0.780) and week 20 (depression: −1.27; 95% CI: −2.79, 0.25; *p* = 0.101, anxiety: −1.26; 95% CI: −2.61, 0.09; *p* = 0.067) (Table 2).

### 3.5 Survival

**FIGURE 3:**
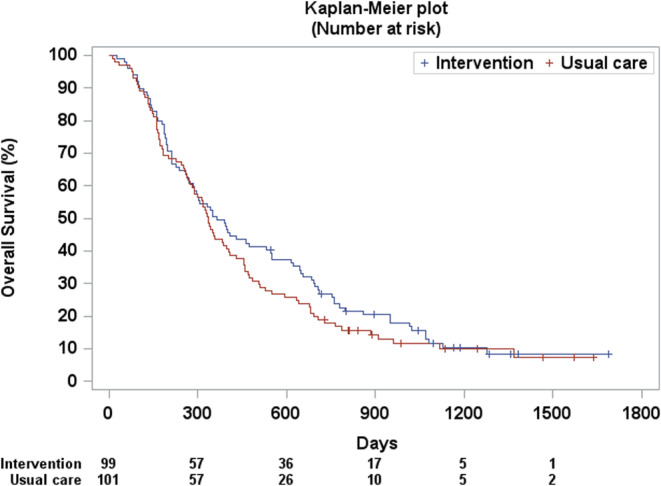
Overall survival.

The 1‐year survival rates in the intervention and usual care groups were 49.5% (95% CI: 39.3, 58.9) and 43.6% (95% CI: 33.8, 52.9), respectively. Survival did not significantly differ between the intervention and usual care groups (median survival time: 12.1 vs. 11.1 months; *p* = 0.302) (Figure 3).

## 4 Discussion

This study examined the effectiveness of a nurse‐led, screening‐triggered, early specialized palliative care intervention program for patients with advanced lung cancer. The results showed no significant differences between the groups during the initial 12 weeks but suggested possible delayed positive effects on multiple outcomes, including QOL and mood, at 20 weeks. Unlike previous studies [2, 3, 4, 5] in which palliative care specialists provided care for all patients, the present intervention combined screening and nurse‐led stepped‐care approaches. We believe our strategy will be easier to adapt in actual clinical practice.

One reason for the lack of significant results could be that some patients in the intervention group did not receive interventions by advanced‐level nurses from the beginning or throughout the entire period based on screening results. Previous studies have often used an intervention model for all patients in the intervention group [2, 3, 4, 5, 7, 8, 9]. Additionally, a relatively high percentage (46.1%) of patients in the usual care group received intervention by professionals belonging to the palliative care team at least once, indicative of the current clinical practice in highly specialized cancer institutions. Therefore, our novel, comprehensive, and feasible intervention strategy should not be abandoned, although further analysis using qualitative research methods is needed to determine which interventions are effective. Another reason may have been the lack of uniform interventions concerning cancer progression and end‐of‐life preparation for all patients in the intervention group. In this study, after building a rapport with the patients, end‐of‐life discussions were postponed until the patients or their family members expressed readiness or willingness to engage in such conversations. Future studies should incorporate advanced care planning to facilitate high‐quality discussions, engaging patients and families in making informed medical decisions [29].

Our study has several limitations. First, blinding was not feasible for the interventions and assessments due to the nature of these interventions. Second, the study was conducted at two highly specialized comprehensive cancer medical institutions. It is possible that these institutions already had palliative care integrated into oncology, potentially reducing the effectiveness of our program. The results might have varied in a more general facility. Finally, we excluded patients with a negative or unknown gene mutation status for which molecular targeted therapy is applicable, as their expected prognosis would differ. This exclusion could introduce a selection bias. Future studies should include this population.

In conclusion, although this trial failed to show the statistical superiority of nurse‐led, screening‐triggered, early specialized palliative care intervention programs over usual care during the 12 weeks, it may have yielded delayed clinical benefits, such as an improvement in QOL. The study design, where some patients in the intervention group received delayed or no intervention, may have diminished the differences between the two groups. Further investigation is needed to identify factors affecting this EPC model and to refine the model as a more effective intervention.

## Author Contributions

**Yoshihisa Matsumoto:** conceptualization (equal), funding acquisition (lead), investigation (lead), methodology (equal), writing – original draft (lead), writing – review and editing (lead). **Shigeki Umemura:** conceptualization (supporting), methodology (supporting), resources (equal), writing – review and editing (supporting). **Ayumi Okizaki:** investigation (supporting), project administration (lead), writing – review and editing (supporting). **Daisuke Fujisawa:** conceptualization (supporting), methodology (supporting), supervision (equal), writing – review and editing (supporting). **Takuhiro Yamaguchi:** formal analysis (lead), supervision (supporting), writing – review and editing (supporting). **Shunsuke Oyamada:** formal analysis (equal), visualization (lead), writing – review and editing (supporting). **Tempei Miyaji:** data curation (lead), project administration (supporting), writing – review and editing (supporting). **Tomoe Mashiko:** data curation (equal), writing – review and editing (supporting). **Naoko Kobayashi:** investigation (equal), writing – review and editing (supporting). **Eriko Satomi:** investigation (supporting), writing – review and editing (supporting). **Daisuke Kiuchi:** investigation (supporting), writing – review and editing (supporting). **Tatsuya Morita:** conceptualization (supporting), methodology (supporting), supervision (equal), writing – review and editing (supporting). **Yosuke Uchitomi:** supervision (equal), writing – review and editing (supporting). **Koichi Goto:** resources (equal), supervision (supporting), writing – review and editing (supporting). **Yuichiro Ohe:** resources (equal), supervision (supporting), writing – review and editing (supporting).

## Conflicts of Interest

Dr. Matsumoto reported grants from the Japan Agency for Medical Research and Development (AMED) (Grants JP16ck0106213 and JP19ck0106502) and the Japan Health, Labour and Welfare Sciences Research Grants (Grant of H27‐Cancer Control‐general‐002). No other disclosures were reported.

## Supporting information


Table S1.

Table S2.

## Data Availability

The datasets used and/or analyzed during the current study are available from the corresponding author upon reasonable request.
